# Supplementary material for: Mobility evaluation by GPS tracking in a rural, low-income population in Cambodia
Source: PLoS One. 2022 May 13;17(5):e0266460. doi: 10.1371/journal.pone.0266460 (PMC9106150; doi:10.1371/journal.pone.0266460)
Supplement: S1 Table — (DOCX) [file pone.0266460.s001.docx]

**S1 Table. Count of logged and analysed GPS points.**

| **Summary** | **Rainy season** | **Dry season** | **All** |
| --- | --- | --- | --- |
| Number of points logged | 1,280,050 | 3,103,532 | 4,383,582 |
| Number of points analysed | 1,044,335 | 1,582,083 | 2,626,418 |
