# Supplementary material for: Mobility evaluation by GPS tracking in a rural, low-income population in Cambodia
Source: PLoS One. 2022 May 13;17(5):e0266460. doi: 10.1371/journal.pone.0266460 (PMC9106150; doi:10.1371/journal.pone.0266460)
Supplement: S2 Table — (DOCX) [file pone.0266460.s002.docx]

**S2 Table: GPS devices’ accuracy controlled as the average error between individual recorded positions and true georeference given by a precise device.**

| Environment | Average accuracy (metres) | Accuracy  standard deviation | % included in  5m radius | % included in  10m radius | % included in  20m radius |
| --- | --- | --- | --- | --- | --- |
| Forest | 11.9 | 9.2 | 22.4 | 50.8 | 88.2 |
| House | 6.1 | 3.1 | 37.9 | 89 | 99.8 |
| Open Space | 5.6 | 6.9 | 51.1 | 95.9 | 99.4 |
| Plantation | 17.2 | 13.3 | 6.6 | 31.1 | 74.8 |
| All | 10.3 | 10.2 | 28.8 | 65.8 | 90.3 |
