# Supplementary material for: Mobility evaluation by GPS tracking in a rural, low-income population in Cambodia
Source: PLoS One. 2022 May 13;17(5):e0266460. doi: 10.1371/journal.pone.0266460 (PMC9106150; doi:10.1371/journal.pone.0266460)
Supplement: S3 Table — (DOCX) [file pone.0266460.s003.docx]

**S3 Table: GPS devices’ precision controlled as the average error between individual recorded positions and centroid of the units’ recorded positions.**

| Environment | Average precision (metres) | Precision  standard deviation | % included in  5m radius | % included in  10m radius | % included in  20m radius |
| --- | --- | --- | --- | --- | --- |
| Forest | 10.7 | 8.5 | 23 | 59.1 | 88.7 |
| House | 5.9 | 3.1 | 37.2 | 88.6 | 99.9 |
| Open Space | 5 | 7.0 | 60.2 | 94.6 | 99.4 |
| Plantation | 15.4 | 10.9 | 9.3 | 36.4 | 77.5 |
| All | 9.4 | 8.9 | 31.5 | 68.9 | 91.1 |
