# Supplementary material for: Mobility evaluation by GPS tracking in a rural, low-income population in Cambodia
Source: PLoS One. 2022 May 13;17(5):e0266460. doi: 10.1371/journal.pone.0266460 (PMC9106150; doi:10.1371/journal.pone.0266460)
Supplement: S4 Table — (DOCX) [file pone.0266460.s004.docx]

**S4 Table: Proportions of tracks belonging to insufficient, poor and optimal categories, allowing selecting optimal GPS tracks for analysis.**

| **Variable** | **Category** | **Rainy season** | | **Dry season** | | **All** | |
| --- | --- | --- | --- | --- | --- | --- | --- |
|  |  | **N** | **%** | **N** | **%** | **N** | **%** |
| Distance | suboptimal | 38 | 15.3 | 42 | 11.7 | 80 | 13.1 |
|  | optimal | 211 | 84.7 | 318 | 88.3 | 529 | 86.9 |
| Duration | suboptimal | 65 | 26.1 | 60 | 16.7 | 125 | 20.5 |
|  | optimal | 184 | 73.9 | 300 | 83.3 | 484 | 79.5 |
| Distance/day | suboptimal | 24 | 9.6 | 76 | 21.1 | 100 | 7.7 |
|  | optimal | 225 | 90.4 | 284 | 78.9 | 509 | 83.6 |
| Track | suboptimal | 85 | 34.1 | 131 | 36.4 | 216 | 35.5 |
|  | optimal | 164 | 65.9 | 229 | 63.6 | 393 | 64.5 |
