# Supplementary material for: Mobility evaluation by GPS tracking in a rural, low-income population in Cambodia
Source: PLoS One. 2022 May 13;17(5):e0266460. doi: 10.1371/journal.pone.0266460 (PMC9106150; doi:10.1371/journal.pone.0266460)
Supplement: S5 Table — Every land use category was compared to the total time logged minus the time in this land use category using a Chi-squared test. According to Bonferonni correction, p-value was significant when p < 0.05/6 = 0.0083. (DOCX) [file pone.0266460.s005.docx]

**S5 Table: Percentage of every land use, speed and time variables attributed to suboptimal and optimal datasets.** Every land use category was compared to the total time logged minus the time in this land use category using a Chi-squared test. According to Bonferonni correction, p-value was significant when p < 0.05/6 = 0.0083.

| Dataset | built-up areas | fields | plantations | forest | slow | fast | night | day |
| --- | --- | --- | --- | --- | --- | --- | --- | --- |
| Suboptimal | 46.4 | 38.7 | 7.3 | 7.5 | 85.5 | 14.5 | 33.6 | 66.4 |
| Optimal | 37.9 | 44.5 | 9.6 | 7.8 | 77.6 | 22.4 | 29.7 | 70.3 |
| p-value | < 10^-6^ | < 10^-6^ | < 10^-6^ | < 10^-6^ | < 10^-6^ | | < 10^-6^ | |
