# Supplementary material for: Mobility evaluation by GPS tracking in a rural, low-income population in Cambodia
Source: PLoS One. 2022 May 13;17(5):e0266460. doi: 10.1371/journal.pone.0266460 (PMC9106150; doi:10.1371/journal.pone.0266460)
Supplement: S6 Table — Discordant data is highlighted in yellow. (DOCX) [file pone.0266460.s006.docx]

**S6 Table: Count of data discrepancies between questionnaire and GPS datasets about participants visits in forest, plantations and fields, for all participants with a complete dataset for questionnaire at week 2 and GPS follow-up (N = 197).** Discordant data is highlighted in yellow.

|  | Questionnaire | |  |
| --- | --- | --- | --- |
| **Land use category** | visiting | not visiting | **GPS data** |
| forest | 133 | 6 | visiting |
|  | 47 | 11 | not visiting |
| plantations | 92 | 29 | visiting |
|  | 48 | 28 | not visiting |
| fields | 95 | 94 | visiting |
|  | 1 | 7 | not visiting |
