# Supplementary material for: Mobility evaluation by GPS tracking in a rural, low-income population in Cambodia
Source: PLoS One. 2022 May 13;17(5):e0266460. doi: 10.1371/journal.pone.0266460 (PMC9106150; doi:10.1371/journal.pone.0266460)
Supplement: S7 Table — (DOCX) [file pone.0266460.s007.docx]

**S7 Table: Total discrepancies between GPS and questionnaire data for participants with a complete dataset (N = 197).**

| Land use category | Value | Discordant data | Concordant data |
| --- | --- | --- | --- |
| forest | N | 53 | 144 |
|  | % | 26.9 | 73.1 |
| plantations | N | 77 | 120 |
|  | % | 39.1 | 60.9 |
| fields | N | 95 | 102 |
|  | % | 48.2 | 51.8 |
